# Bis(acetato-κ*O*)bis­(4,5-dimethyl­benzene-1,2-diamine-κ*N*)zinc

**DOI:** 10.1107/S160053681203036X

**Published:** 2012-07-10

**Authors:** David K. Geiger

**Affiliations:** a Department of Chemistry, State University of New York – College at Geneseo, 1 College Circle, Geneseo, NY 14454, USA

## Abstract

The structure of the title compound, [Zn(CH_3_COO)_2_(C_8_H_12_N_2_)_2_], has one half molecule in the asymmetric unit. The Zn^II^ atom is situated on a twofold rotation axis and is tetrahedrally coordinated by two N and two O atoms. The crystal packing displays inter­molecular N—H⋯O hydrogen bonds and intra­molecular N—H⋯O and N—H⋯N hydrogen bonding.

## Related literature

For the role of complexes in biochemical systems with zinc in tetrahedral coordination, see: Parkin (2004 ▶); Maret & Li (2009 ▶). For the structure of the corresponding 1,2-diamino­benzene complex, see: Mei *et al.* (2009 ▶). For an example of a structurally characterized tetramine complex with zinc in tetrahedral coordination, see: Xu *et al.* (1998 ▶). For an example carboxyl­ate coordination in a similar complex, see: Harding (2001 ▶).
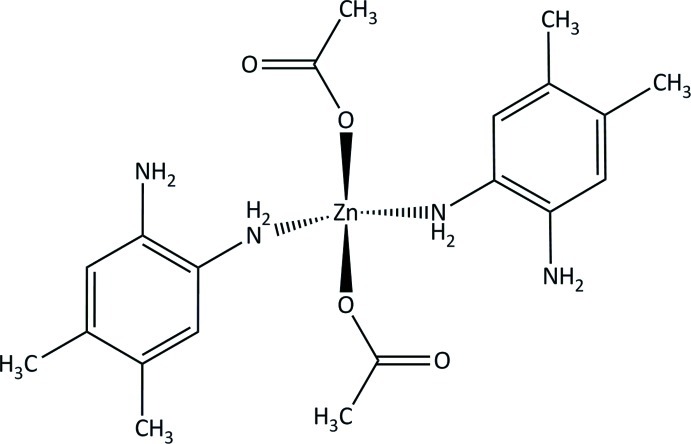


## Experimental

### 

#### Crystal data

[Zn(C_2_H_3_O_2_)_2_(C_8_H_12_N_2_)_2_]
*M* *_r_* = 455.85
Monoclinic, 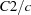

*a* = 18.432 (3) Å
*b* = 4.7414 (6) Å
*c* = 25.740 (4) Å
β = 92.284 (4)°
*V* = 2247.8 (5) Å^3^
*Z* = 4
Mo *K*α radiation
μ = 1.12 mm^−1^
*T* = 200 K
0.80 × 0.30 × 0.20 mm

#### Data collection

Bruker SMART X2S benchtop diffractometer
Absorption correction: multi-scan (*SADABS*; Sheldrick, 2008*b* ▶) *T*_min_ = 0.467, *T*_max_ = 0.806
11403 measured reflections
1986 independent reflections
1905 reflections with *I* > 2σ(*I*)
*R*_int_ = 0.046

#### Refinement

*R*[*F* ^2^ > 2σ(*F* ^2^)] = 0.036
*wR*(*F* ^2^) = 0.094
*S* = 1.12
1986 reflections
151 parameters
H atoms treated by a mixture of independent and constrained refinement
Δρ_max_ = 0.67 e Å^−3^
Δρ_min_ = −0.37 e Å^−3^

### 

Data collection: *APEX2* (Bruker, 2010 ▶); cell refinement: *SAINT* (Bruker, 2009 ▶); data reduction: *SAINT*; program(s) used to solve structure: *SHELXS97* (Sheldrick, 2008*a* ▶); program(s) used to refine structure: *SHELXL97* (Sheldrick, 2008*a* ▶); molecular graphics: *XSHELL* (Bruker, 2004 ▶) and *Mercury* (Macrae *et al.*, 2008 ▶); software used to prepare material for publication: *publCIF* (Westrip, 2010 ▶).

## Supplementary Material

Crystal structure: contains datablock(s) I, global. DOI: 10.1107/S160053681203036X/bv2208sup1.cif

Supplementary material file. DOI: 10.1107/S160053681203036X/bv2208Isup2.cdx

Structure factors: contains datablock(s) I. DOI: 10.1107/S160053681203036X/bv2208Isup3.hkl

Additional supplementary materials:  crystallographic information; 3D view; checkCIF report

## Figures and Tables

**Table 1:** Hydrogen-bond geometry (Å, °)

*D*—H⋯*A*	*D*—H	H⋯*A*	*D*⋯*A*	*D*—H⋯*A*
N1—H1*A*⋯O1^i^	0.84 (4)	2.10 (4)	2.897 (3)	158 (3)
N2—H2*A*⋯O2	0.88 (3)	2.15 (3)	3.013 (3)	168 (3)
N2—H2*B*⋯N2^ii^	0.81 (4)	2.26 (4)	3.076 (3)	179 (3)

Symmetry codes: (i) 
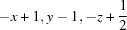
; (ii) 
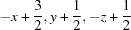
.

**Fig. 1.**
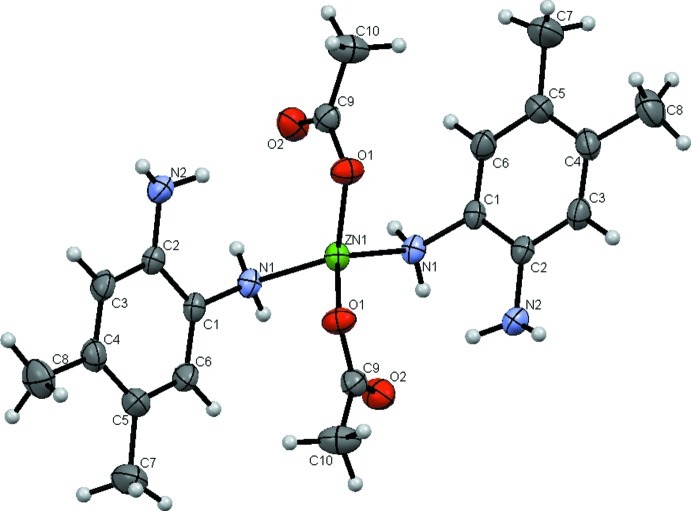
Perspective view of the title compound with displacement ellipsoids of non-hydrogen atoms drawn at the 50% probability level.

**Fig. 2.**
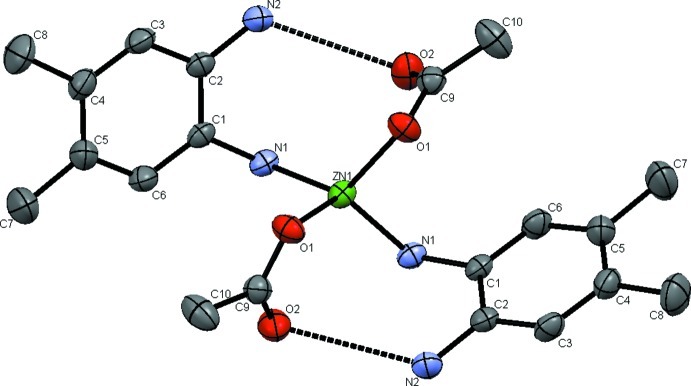
Perspective view of the title compound displaying the intramolecular hydrogen bonding.

**Fig. 3.**
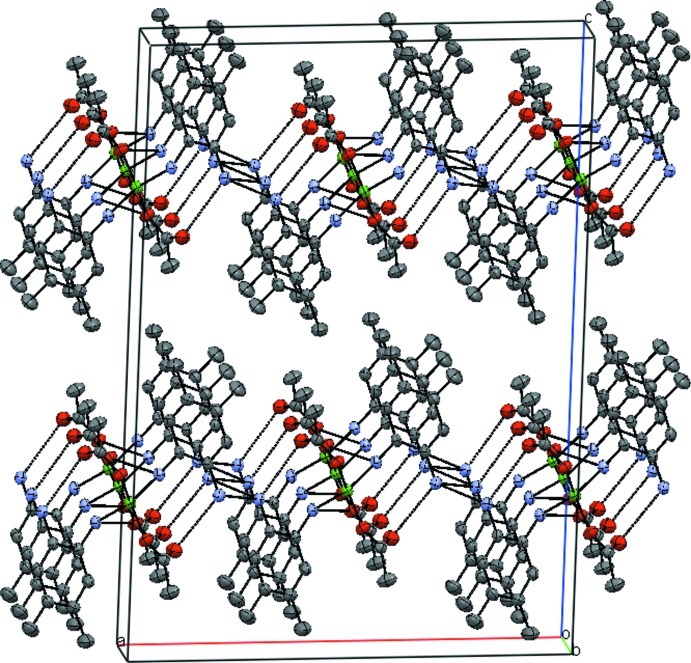
View of the unit cell of the title compound down the b axis displaying the intermolecular hydrogen bonding network.

## supplementary crystallographic information

### Comment

Tetrahedrally coordinated zinc complexes play important structural (*e.g.*, zinc fingers) and catalytic (*e.g.*, carbonic anydrase) roles in biochemical systems (Parkin 2004, Maret & Li 2009). Although coordination *via* three amino acid residues (Zn—N coordination) and a water or hydroxide ligand is the most common coordination motif, carboxylate coordination is also known (Harding 2001). The title compound exhibits tetrahedral coordination involving two phenylenediamine ligands and two acetate ligands all coordinated in a monodentate fashion (see figure 1). The Zn atom sits on a twofold rotation axis resulting in a one-half molecule asymmetric unit. The complex exhibits intramolecular hydrogen bonding involving the uncoordinated amine nitrogen, N2, and the uncoordinated acetate oxygen, O2 (see figure 2). In addition, one of the H atoms of the coordinated amine is involved in two weak intramolecular hydrogen bonding intractions with the uncoordinated acetate oxygen atom (N1—H1B^···^O2 = 2.77 (3) Å, 114 (3)°) and the coordinated amine (N1—H1B^···^N2 = 2.57 (3) Å, 99 (3)°). An intermolecular hydrogen bonding network involving N2—H^···^N2 and N1—H^···^O1 interactions results in planes of molecules perpindicular to the *c* axis (see figure 3).

### Experimental

The title compound was prepared by the reaction of two equivalents of 4,5-dimethyl-1,2-diaminobenzene with zinc(II) acetate dihydrate in refluxing ethanol. Slow evaporation of the solvent resulted in large, well formed crystals. The sample used for analysis was cut from a larger crystal. ^1^H NMR spectrum (CDCl_3_, 400 MHz, p.p.m.): 6.15 (4*H*, s), 3.89 (8*H*, b), 2.05 (12*H*, s), 1.92 (6*H*, s).

### Refinement

The structure was originally solved in the non-centrosymmetric space group *Cc* because the mean |E*E-1| statistic was 0.745. The structure refined to *R*_1_ = 0.051. However, many atoms displayed disc-shaped thermal ellipsoids and one of the nitrogen atoms coordinated to the zinc became nonpositive definite. Inverting the structure gave no improvement. Using TWIN resulted in a refined BASF of 0.49 with no significant improvement in the *R*_1_ value (0.048) or thermal parameters (the nitrogen remained nonpositive definite). The structure was subsequently solved and successfully refined in the centrosymmetric space group *C*2/*c*, which resulted in a lower *R*_1_ (0.0363)and much improved behavior of the thermal parameters. All H atoms atoms were found in difference fourier maps. Hydrogen atoms bonded to carbon atoms were refined using a riding model (AFIX 43 for aromatic C—H and AFIX 137 for methyl groups). The atomic coordinates and isotropic thermal parameters of all amine hydrogen atoms were refined.

### Crystal data

**Table:**

[Zn(C_2_H_3_O_2_)_2_(C_8_H_12_N_2_)_2_]	*F*(000) = 960
*M**_r_* = 455.85	*D*_x_ = 1.347 Mg m^−^^3^
Monoclinic, *C*2/*c*	Mo *K*α radiation, λ = 0.71073 Å
Hall symbol: -C2yc	Cell parameters from 5876 reflections
*a* = 18.432 (3) Å	θ = 2.7–25.0°
*b* = 4.7414 (6) Å	µ = 1.12 mm^−^^1^
*c* = 25.740 (4) Å	*T* = 200 K
β = 92.284 (4)°	Plate, colourless
*V* = 2247.8 (5) Å^3^	0.80 × 0.30 × 0.20 mm
*Z* = 4	

### Data collection

**Table:**

Bruker SMART X2S benchtop diffractometer	1986 independent reflections
Radiation source: fine-focus sealed tube	1905 reflections with *I* > 2σ(*I*)
Graphite monochromator	*R*_int_ = 0.046
ω scans	θ_max_ = 25.0°, θ_min_ = 2.7°
Absorption correction: multi-scan (*SADABS*; Sheldrick, 2008*b*)	*h* = −21→21
*T*_min_ = 0.467, *T*_max_ = 0.806	*k* = −5→5
11403 measured reflections	*l* = −28→30

### Refinement

**Table:**

Refinement on *F*^2^	Primary atom site location: structure-invariant direct methods
Least-squares matrix: full	Secondary atom site location: difference Fourier map
*R*[*F*^2^ > 2σ(*F*^2^)] = 0.036	Hydrogen site location: inferred from neighbouring sites
*wR*(*F*^2^) = 0.094	H atoms treated by a mixture of independent and constrained refinement
*S* = 1.12	*w* = 1/[σ^2^(*F*_o_^2^) + (0.0434*P*)^2^ + 4.1592*P*] where *P* = (*F*_o_^2^ + 2*F*_c_^2^)/3
1986 reflections	(Δ/σ)_max_ < 0.001
151 parameters	Δρ_max_ = 0.67 e Å^−^^3^
0 restraints	Δρ_min_ = −0.37 e Å^−^^3^

### Special details

**Table:**

Geometry. All e.s.d.'s (except the e.s.d. in the dihedral angle between two l.s. planes) are estimated using the full covariance matrix. The cell e.s.d.'s are taken into account individually in the estimation of e.s.d.'s in distances, angles and torsion angles; correlations between e.s.d.'s in cell parameters are only used when they are defined by crystal symmetry. An approximate (isotropic) treatment of cell e.s.d.'s is used for estimating e.s.d.'s involving l.s. planes.
Refinement. Refinement of *F*^2^ against ALL reflections. The weighted *R*-factor *wR* and goodness of fit *S* are based on *F*^2^, conventional *R*-factors *R* are based on *F*, with *F* set to zero for negative *F*^2^. The threshold expression of *F*^2^ > σ(*F*^2^) is used only for calculating *R*-factors(gt) *etc*. and is not relevant to the choice of reflections for refinement. *R*-factors based on *F*^2^ are statistically about twice as large as those based on *F*, and *R*- factors based on ALL data will be even larger.

### Fractional atomic coordinates and isotropic or equivalent isotropic displacement parameters (Å^2^)

**Table:**

	*x*	*y*	*z*	*U*_iso_*/*U*_eq_	
Zn1	0.5000	0.21781 (8)	0.2500	0.02608 (16)	
O1	0.53194 (10)	0.4948 (4)	0.30352 (6)	0.0343 (4)	
O2	0.60458 (11)	0.1492 (4)	0.33024 (7)	0.0421 (5)	
N1	0.56934 (12)	−0.0427 (5)	0.21196 (8)	0.0281 (5)	
N2	0.69849 (13)	0.2531 (6)	0.23902 (9)	0.0360 (6)	
C1	0.60972 (12)	0.0974 (5)	0.17273 (9)	0.0262 (5)	
C2	0.67121 (13)	0.2524 (5)	0.18723 (10)	0.0279 (6)	
C3	0.70681 (14)	0.3968 (6)	0.14856 (10)	0.0344 (6)	
H3	0.7496	0.4999	0.1579	0.041*	
C4	0.68215 (14)	0.3954 (7)	0.09706 (10)	0.0364 (6)	
C5	0.61975 (15)	0.2423 (7)	0.08302 (10)	0.0383 (7)	
C6	0.58503 (14)	0.0931 (7)	0.12132 (10)	0.0351 (6)	
H6	0.5431	−0.0149	0.1119	0.042*	
C7	0.5892 (2)	0.2368 (9)	0.02749 (12)	0.0659 (12)	
H7A	0.5444	0.1254	0.0258	0.099*	
H7B	0.6248	0.1515	0.0049	0.099*	
H7C	0.5787	0.4298	0.0159	0.099*	
C8	0.72326 (19)	0.5613 (8)	0.05768 (12)	0.0553 (9)	
H8A	0.7708	0.6160	0.0729	0.083*	
H8B	0.6957	0.7310	0.0478	0.083*	
H8C	0.7300	0.4446	0.0268	0.083*	
C9	0.57583 (14)	0.3768 (6)	0.33792 (9)	0.0308 (6)	
C10	0.58890 (18)	0.5363 (7)	0.38778 (11)	0.0488 (8)	
H10A	0.6249	0.4360	0.4099	0.073*	
H10B	0.5433	0.5516	0.4059	0.073*	
H10C	0.6070	0.7256	0.3801	0.073*	
H1A	0.5413 (19)	−0.167 (7)	0.1990 (12)	0.045 (9)*	
H1B	0.5973 (17)	−0.120 (7)	0.2345 (12)	0.039 (8)*	
H2A	0.6658 (18)	0.234 (6)	0.2628 (12)	0.039 (9)*	
H2B	0.7263 (18)	0.383 (8)	0.2448 (12)	0.042 (9)*	

### Atomic displacement parameters (Å^2^)

**Table:**

	*U*^11^	*U*^22^	*U*^33^	*U*^12^	*U*^13^	*U*^23^
Zn1	0.0257 (2)	0.0218 (2)	0.0310 (2)	0.000	0.00465 (15)	0.000
O1	0.0391 (10)	0.0294 (10)	0.0338 (9)	0.0044 (8)	−0.0066 (7)	−0.0022 (8)
O2	0.0462 (12)	0.0404 (13)	0.0398 (10)	0.0137 (10)	0.0034 (8)	0.0026 (9)
N1	0.0235 (10)	0.0279 (13)	0.0330 (11)	−0.0022 (10)	0.0030 (9)	0.0003 (9)
N2	0.0251 (11)	0.0492 (17)	0.0336 (12)	−0.0046 (11)	−0.0011 (10)	0.0021 (10)
C1	0.0210 (11)	0.0258 (13)	0.0323 (12)	0.0021 (10)	0.0053 (9)	−0.0003 (10)
C2	0.0213 (12)	0.0306 (15)	0.0319 (12)	0.0030 (10)	0.0022 (9)	−0.0002 (10)
C3	0.0232 (12)	0.0393 (16)	0.0410 (14)	−0.0057 (12)	0.0055 (10)	−0.0013 (12)
C4	0.0315 (14)	0.0424 (17)	0.0360 (13)	0.0002 (13)	0.0101 (11)	0.0045 (12)
C5	0.0327 (14)	0.0525 (19)	0.0297 (13)	−0.0007 (13)	0.0026 (11)	0.0009 (12)
C6	0.0260 (13)	0.0440 (17)	0.0355 (13)	−0.0073 (12)	0.0033 (10)	−0.0067 (12)
C7	0.053 (2)	0.110 (4)	0.0338 (16)	−0.014 (2)	−0.0010 (14)	0.0017 (18)
C8	0.0550 (19)	0.066 (2)	0.0455 (17)	−0.0125 (18)	0.0163 (14)	0.0110 (16)
C9	0.0298 (13)	0.0311 (15)	0.0314 (12)	−0.0015 (11)	0.0017 (10)	0.0037 (11)
C10	0.066 (2)	0.0427 (19)	0.0368 (15)	0.0084 (16)	−0.0131 (14)	−0.0038 (13)

### Geometric parameters (Å, º)

**Table:**

Zn1—O1	1.9759 (18)	C3—H3	0.9500
Zn1—O1^i^	1.9759 (18)	C4—C5	1.395 (4)
Zn1—N1	2.054 (2)	C4—C8	1.511 (4)
Zn1—N1^i^	2.054 (2)	C5—C6	1.390 (4)
O1—C9	1.302 (3)	C5—C7	1.516 (4)
O2—C9	1.222 (3)	C6—H6	0.9500
N1—C1	1.441 (3)	C7—H7A	0.9800
N1—H1A	0.84 (4)	C7—H7B	0.9800
N1—H1B	0.84 (3)	C7—H7C	0.9800
N2—C2	1.406 (3)	C8—H8A	0.9800
N2—H2A	0.88 (3)	C8—H8B	0.9800
N2—H2B	0.81 (4)	C8—H8C	0.9800
C1—C6	1.382 (3)	C9—C10	1.501 (4)
C1—C2	1.389 (4)	C10—H10A	0.9800
C2—C3	1.394 (4)	C10—H10B	0.9800
C3—C4	1.384 (4)	C10—H10C	0.9800
			
O1—Zn1—O1^i^	96.70 (10)	C6—C5—C4	118.6 (2)
O1—Zn1—N1	123.94 (8)	C6—C5—C7	119.7 (3)
O1^i^—Zn1—N1	103.95 (8)	C4—C5—C7	121.7 (3)
O1—Zn1—N1^i^	103.95 (8)	C1—C6—C5	121.9 (2)
O1^i^—Zn1—N1^i^	123.94 (8)	C1—C6—H6	119.1
N1—Zn1—N1^i^	106.06 (13)	C5—C6—H6	119.1
C9—O1—Zn1	110.40 (17)	C5—C7—H7A	109.5
C1—N1—Zn1	113.93 (17)	C5—C7—H7B	109.5
C1—N1—H1A	112 (2)	H7A—C7—H7B	109.5
Zn1—N1—H1A	103 (2)	C5—C7—H7C	109.5
C1—N1—H1B	111 (2)	H7A—C7—H7C	109.5
Zn1—N1—H1B	108 (2)	H7B—C7—H7C	109.5
H1A—N1—H1B	109 (3)	C4—C8—H8A	109.5
C2—N2—H2A	115 (2)	C4—C8—H8B	109.5
C2—N2—H2B	112 (2)	H8A—C8—H8B	109.5
H2A—N2—H2B	113 (3)	C4—C8—H8C	109.5
C6—C1—C2	119.9 (2)	H8A—C8—H8C	109.5
C6—C1—N1	120.3 (2)	H8B—C8—H8C	109.5
C2—C1—N1	119.6 (2)	O2—C9—O1	122.1 (2)
C1—C2—C3	118.1 (2)	O2—C9—C10	121.8 (2)
C1—C2—N2	120.9 (2)	O1—C9—C10	116.1 (2)
C3—C2—N2	121.0 (2)	C9—C10—H10A	109.5
C4—C3—C2	122.4 (2)	C9—C10—H10B	109.5
C4—C3—H3	118.8	H10A—C10—H10B	109.5
C2—C3—H3	118.8	C9—C10—H10C	109.5
C3—C4—C5	119.1 (2)	H10A—C10—H10C	109.5
C3—C4—C8	119.1 (3)	H10B—C10—H10C	109.5
C5—C4—C8	121.8 (3)		

Symmetry code: (i) −*x*+1, *y*, −*z*+1/2.

### Hydrogen-bond geometry (Å, º)

**Table:**

*D*—H···*A*	*D*—H	H···*A*	*D*···*A*	*D*—H···*A*
N1—H1*A*···O1^ii^	0.84 (4)	2.10 (4)	2.897 (3)	158 (3)
N2—H2*A*···O2	0.88 (3)	2.15 (3)	3.013 (3)	168 (3)
N2—H2*B*···N2^iii^	0.81 (4)	2.26 (4)	3.076 (3)	179 (3)

Symmetry codes: (ii) −*x*+1, *y*−1, −*z*+1/2; (iii) −*x*+3/2, *y*+1/2, −*z*+1/2.
